# First record of Risso's dolphin *Grampus griseus* (Cuvier, 1812) in Icelandic waters

**DOI:** 10.1002/ece3.10477

**Published:** 2023-08-31

**Authors:** Valérie Chosson, Haseeb S. Randhawa, Guðjón M. Sigurðsson, Sverrir D. Halldórsson, Þorvaldur Þ. Björnsson, Vilhjálmur Svansson, Sandra M. Granquist, Karl Gunnarsson, Filipa I. P. Samarra, Christophe Pampoulie

**Affiliations:** ^1^ Marine and Freshwater Research Institute Hafnarfjörður Iceland; ^2^ Faculty of Life and environmental Sciences University of Iceland Reykjavik Iceland; ^3^ South Atlantic Environmental Research Institute Stanley Falkland Islands; ^4^ New Brunswick Museum Saint John New Brunswick Canada; ^5^ Icelandic Institute of Natural History Garðabær Iceland; ^6^ The Institute of Experimental Pathology University of Iceland Reykjavík Iceland; ^7^ The Icelandic Seal Center Hvammstangi Iceland; ^8^ University of Iceland's Institute of Research Centres Vestmannaeyjar Iceland

**Keywords:** delphinidae, iceland, necropsy, North Atlantic, stranding

## Abstract

In July 2022, two Risso's dolphins were reported stranded in Hrútafjörður (N65° 09,503; W21° 05,529), a fjord in northern Iceland. These events represent the first confirmed observations and strandings of Risso's dolphins in Icelandic waters. Given the uniqueness of these events, a decision was made to conduct full necropsies on these individuals. This study reports findings from viral and parasitological investigations, morphological and fitness measurements, as well as stomach and intestine content analysis for each of the Risso's dolphin specimens. The results of the necropsies do not suggest any other cause of death than lack of food and exhaustion. A large plastic fragment in one individual's stomach supports these suggestions. The presence of those specimens in the middle of the subarctic ocean illustrates ongoing changes in spatial distribution expanding northward, impacting not only Risso's dolphins but more generally marine life and biodiversity.

## Graphical abstract

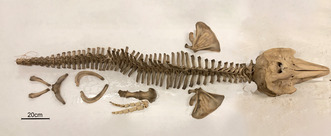

The Risso's dolphin *Grampus griseus* (Cuvier 1812) is widely distributed in tropical and temperate waters of both the north and south hemispheres (Baird, 2009). In the North Atlantic Ocean, Risso's dolphins have been observed from the equator to as far north as 64° N (Bolch et al., 2012; Jefferson et al., 2014). In recent years observations at 69° N latitude in coastal northern Norway have been regular (Alexander Eckerle, personal communication; Figure 1). On the eastern margin of the North Atlantic Ocean Basin, Risso's dolphins have also been regular visitors in the Shetland Islands, Scotland, the North Sea, and the Faroe Islands since 2009 (Bolch et al., 2012). Despite being classified as “Least Concern” by the ICUN, limited information is available for Risso's dolphins (Kiszka & Braulik, 2018).

**FIGURE 1:**
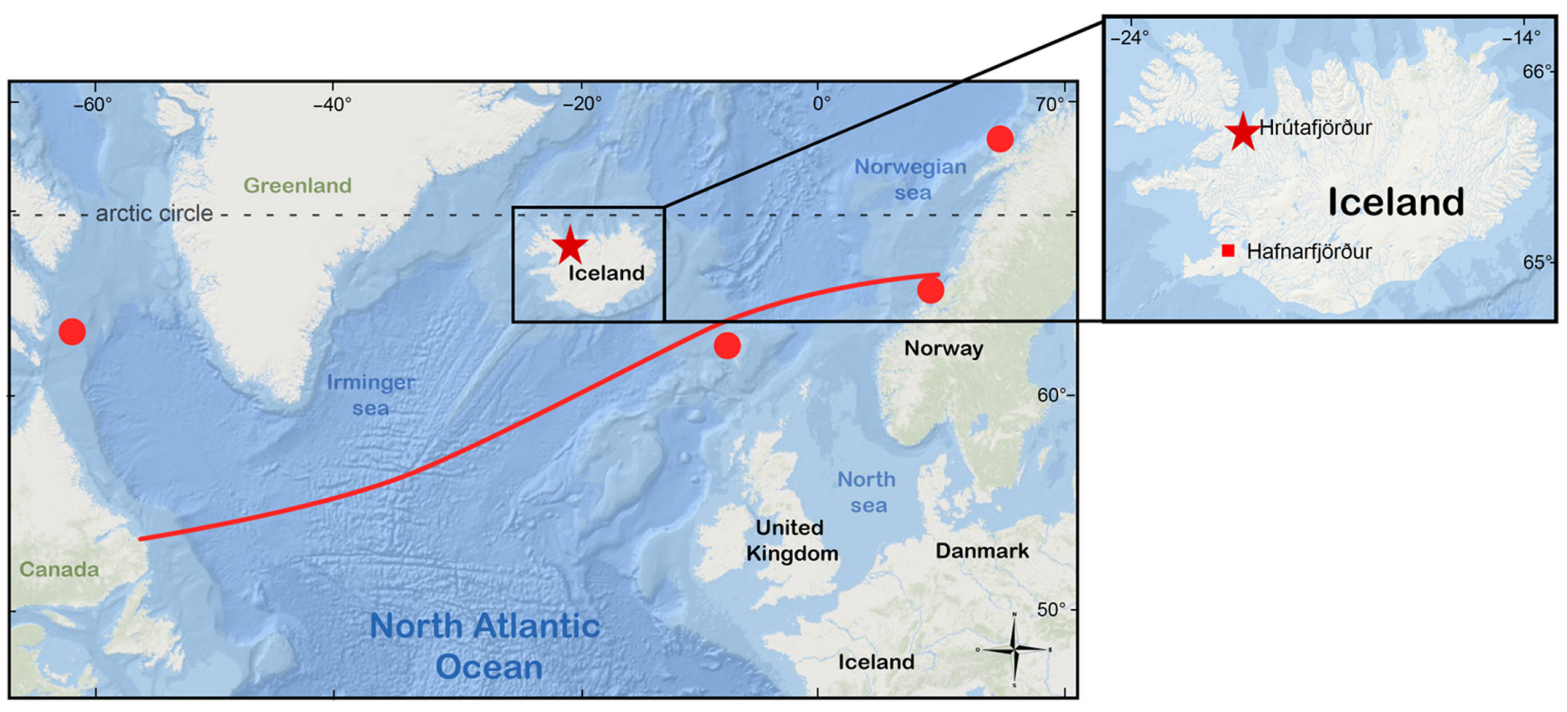
Risso's dolphin distribution in the North Atlantic Ocean. The red line shows the northern limit of the distribution observed in the North Atlantic (Jefferson et al., 2014). The dashed gray line shows the position of the Arctic Circle latitude (N66°50). The red star shows the location of the stranding events S2214‐1 and S2214‐2 reported in Iceland. Red dots show the location of Risso's dolphins' observations outside their normal distribution. In the right panel, the red star shows a more precise location of the stranding events in the north of Iceland and the red square shows the location of the necropsy facilities.

On the 12th of July 2022, one dead female Risso's dolphin (stranding ID S2214‐1) was reported stranded in Hrútafjörður (N65° 09,503 W21° 05,529), a fjord in northern Iceland (Figure 1). At the time of the stranding report, a second individual (a male) was reported alive but in poor condition, swimming in the vicinity of the dead stranded female. After close monitoring and several unsuccessful attempts to resurface the male (stranding ID S2214‐2), the local chief veterinarian decided to euthanize it on the 14th of July 2022.

Stranding and bycatch events have been systematically recorded by the Marine and Freshwater Research Institute of Iceland (MFRI) since 1982 (strandings) and 1992 (bycatch) and annually reported to the North Atlantic Marine Mammal Commission (NAMMCO) since 1992. So far, no Risso's dolphin has been reported. These events, therefore, represent the first confirmed observations and strandings of Risso's dolphins in Icelandic waters. Given the uniqueness of these events, a decision was made to transport the animals to MFRI headquarters in Hafnarfjörður, southwest Iceland (Figure 1), where facilities and expertise were available to conduct full necropsies.

The specimens were stored frozen at −25°C from the 27th of July 2022 to the 5th and 6th of December 2022 (Table 1), when scientists from the University of Iceland, University of Iceland's Institute of Research Centers, the Institute of Experimental Pathology, the MFRI, and the Icelandic Institute of Natural History performed a complete necropsy of both specimens. External morphological features were assessed using pictures (Figure 2) taken on the stranding site following the stranding event.

**TABLE 1:** Timeline between the first observation of the individuals, their storage at −25°C, and the records of Body Condition Score (Joblon et al., 2014) and skin stage (Hartman et al., 2015).

Risso's dolphin specimen	S2214‐1	S2214‐2
Sex	Female	Male
Date of the first live observation	–	12/07/2022
Date of last live observation	–	14/07/2022
Date of death	<12/07/2022	14/07/2022
Body condition score	BSC‐1	BSC‐1
Skin stage	4 (severe scarification)	2 (limited scarification)
Total fresh body length (cm)	276	NA
Date of freezing	27/07/2022	27/07/2022
Necropsy date	06/12/2022	05/12/2022

Abbreviation: NA, nonavailable.

**FIGURE 2:**
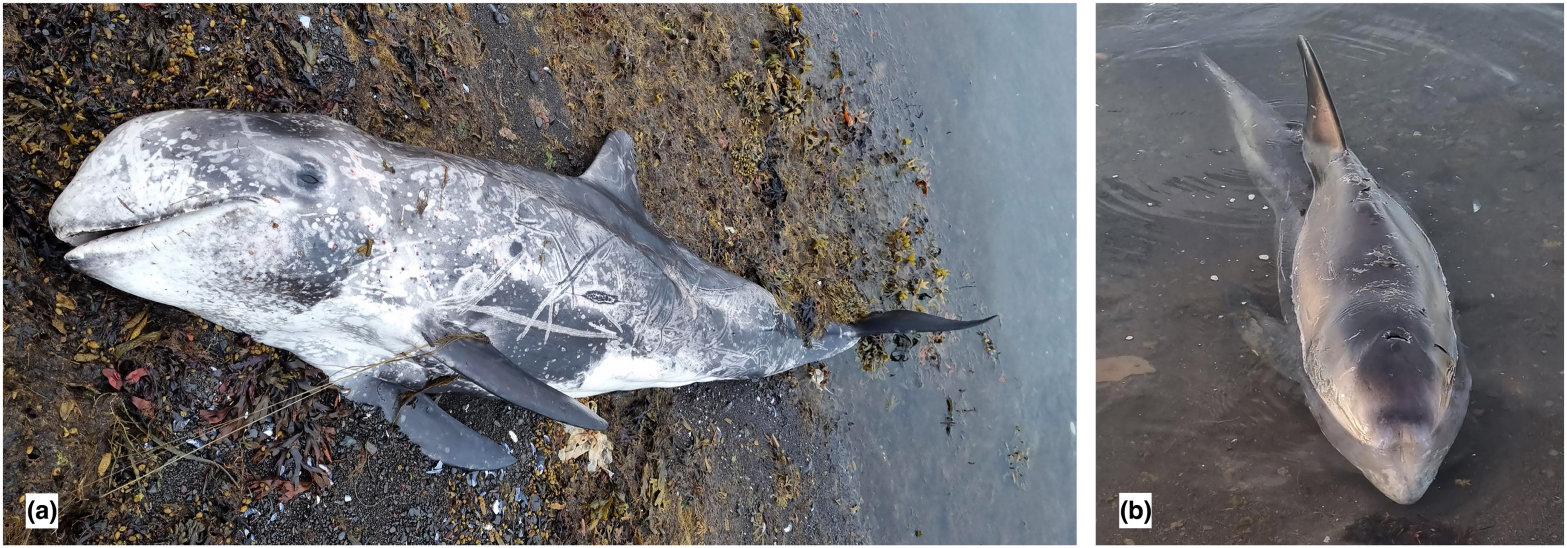
Pictures featuring both animals taken on the 12th of July 2022 on the stranding site (Figure 1). (a) S2214‐1 an adult female and (b) S2214‐2 a juvenile male.

The body condition score (BCS), based on the system for delphinids (Joblon et al., 2014) was used to assess the nutritional status of the individuals at the time of stranding. Both individuals were considered emaciated (BCS = 1). Following Hartman et al. (2015) protocol, the age range of the individuals was determined based on skin characteristics, such as coloration and intensity of scarification in specific body areas (head, frontal back, saddle, and dorsal fin). The female (Figure 2a), which featured overlapping scars with original skin still visible on the head area, isolated scars on the dorsal fin and the frontal back, ranked as adult female with severe scarification (rank 4). The male's skin (Figure 2b) exhibited a general body coloration from pale gray to dark brown with isolated scars on the head, including cephalopod scars, which ranked it as a juvenile with a limited scarification (rank 2; see Table 1).

Morphometric measurements were taken on the individuals once thawed and prior to the necropsies being conducted (Table 2). For the male (S2214‐2), external measurements of the upper head were not taken, due to the skull's damage during the euthanizing procedure and transportation to the freezer facilities. For the female (S2214‐1), measurements involving the lower ventral part were not taken as this part of the body was damaged. This damage was likely caused by scavengers during the postmortem days spent on land.

**TABLE 2:** Morphometric measurements (cm) from the Risso's dolphins S2214‐1 and S2214‐2 at the time of necropsy.

	S2214‐1	S2214‐2
Total fresh body length	276	NA
Length at necropsy	260	222
Sex	Female (adult)	Male (juvenile)
Length from Snout to		
Anterior to blow hole	31	29
Anterior to Melon	12	NA
Anterior to eye	29	25
Commissure	25	NA
Ear	41	NA
Anterior to flipper insertion	53	NA
Posterior to flipper insertion	61	NA
Anterior to Dorsal fin insertion	117	99
Posterior to Dorsal fin insertion	149	132
Fluke's notch	260	222
Anus center	NA	142
Genital slit's center	NA	123

Abbreviation: NA, nonavailable.

To complement the body condition evaluation (Table 1), the half girth (from dorsal midline to ventral midline) and blubber thickness of both individuals were measured. The half girth was measured between the dorsal line and the center of the ventral line in five different locations (labeled 1–5) along the body (Figure 3).

**FIGURE 3:**
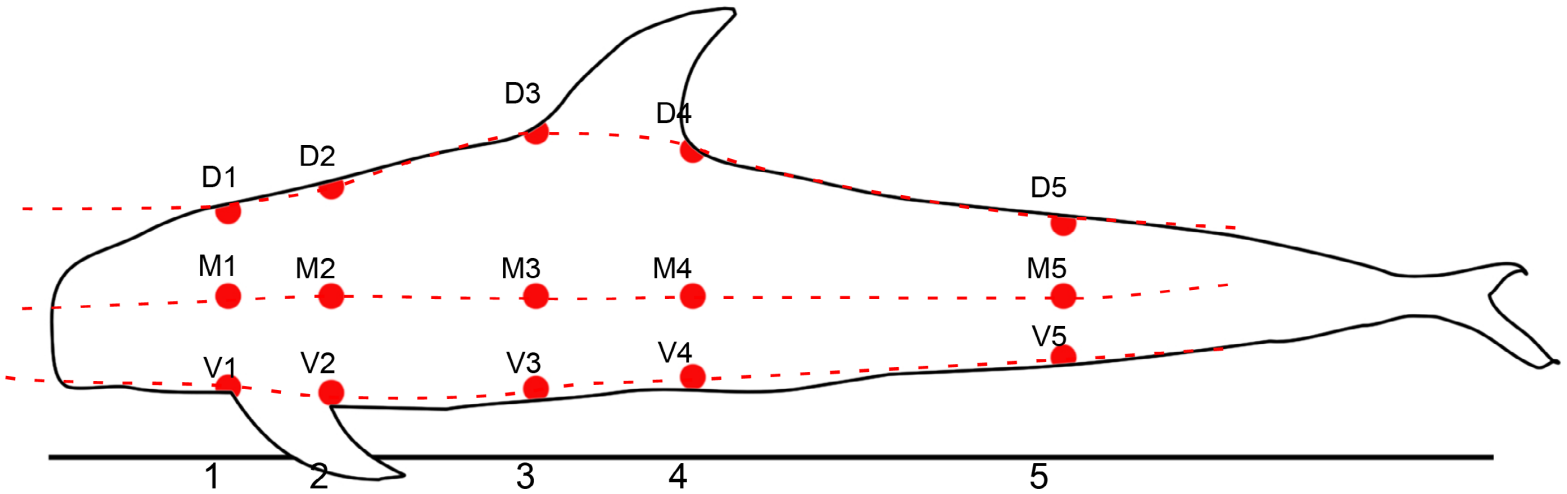
Measurement and sampling positions collected during the necropsy (Lockyer & Waters, 1986). Half girth measurements were taken on position 1 (anterior to the flipper insertion), 2 (posterior to the flipper insertion), 3 (anterior to the dorsal fin insertion), 4 (posterior to the dorsal fin insertion), and 5 (midtail position). The blubber thickness was measured along the dorsal median line (from D1 to D5), along the ventral line (V1 to V5), and along the midline (M1 to M5) of the dolphin's body.

The body condition of both individuals was considered emaciated (BCS = 1; see Joblon et al., 2014, Table 1). These results were supported by girth and blubber thickness measurements (Table 3). The female (S2214‐1) had blubber thickness down to less than 1 cm in the area between M2 and M3 (Figure 3 and Table 3). The male (S2214‐2) also presented very thin blubber, down to 1.5 cm (Figure 3 and Table 3).

**TABLE 3:** Half girth measurements from dorsal midline to ventral midline and blubber thickness at 15 locations, dorsal (D1 to D5), medium (M1 to M5) and ventral (V1 to V5) (Table 2, Figure 3).

Individuals	Half girth (cm)					Blubber thickness (cm)														
						On dorsal line					On midline					On ventral line				
	G1	G2	G3	G4	G5	D1	D2	D3	D4	D5	M1	M2	M3	M4	M5	V1	V2	V3	V4	V5
S2214‐1	53	53	53	45	28	2.6	1.9	2.2	NA	2.4	2.0	0.7	0.6	1.5	1.8	NA	NA	NA	NA	NA
S2214‐2	54	55	52	37	24	4.0	2.0	2.5	3.0	3.5	2.5	2.0	2.5	2.0	1.5	3.0	2.0	2.0	1.5	2.5

Abbreviation: NA, Nonavailable.

Molecular confirmation of the identification as Risso's dolphin was conducted by extracting DNA from tissue samples using the fish buffer technique described in Devlin et al. (2004). Mitochondrial DNA (3′ end of the cytochrome b gene to 5′ end of the D‐loop region) was amplified from both samples using *Taq* 2× Master Mix (25 μL volume) and PCR primers M.whale‐PCR‐F‐b (5′‐GAT CGG TGG CCA ACC CGT AGA AC‐3′) and MW‐PCR‐R (5′‐GGT CCT GAA GTA AGA ACC AGA TG‐3′; see Pampoulie et al., 2021). PCR volumes consisted of 12.50 μL Master Mix, 0.35 μL of each primer (50 μM), 0.5 μL template DNA, and 11.30 μL MQH_2_O and fragments were amplified according to protocol described in Pampoulie et al. (2021). PCR products were purified using ExoSap PCR presequencing purification kits (Applied Biosystems). Sequence data were generated using the aforementioned PCR primers via Sanger sequencing of amplicons by capillary electrophoresis performed by Azenta (Genewiz; Leipzig, Germany). Sequence data were visualized, edited, and contigs assembled using Sequencher 5.4.6™ (GeneCodes) and screened using BLASTn (McGinnis & Madden, 2004).

The identity of both samples was genetically confirmed as Risso's dolphins with 100% overlapping of their mitochondrial DNA (partial 3′ cytochrome b gene to partial 5′ end of the control region; mtDNA, 599 bp) to sequences available in NCBI. Sequences for SS214‐1 and SS214‐2 (NCBI accession numbers OR381458 and OR381459, respectively) were identical and both shared 99.33%, 99.00%, and 98.83% sequence similarity to NC_012062, LC630882, and AB018584, respectively.

During necropsy, all organs were collected and weighed, whenever possible (Table 4). However, due to the time elapsed between their respective time of death and the day when they were frozen (Table 1), enzyme digestive processes had already started to affect some tissues and organs, limiting the samples that could be collected.

**TABLE 4:** Weight (g) of organs from the Risso's dolphins S2214‐1 and S2214‐2.

Individuals	Organ's weight (g)							
	Heart^a^	Liver	Kidneys	Lungs	Spleen	Pancreas	Gonads	Stomach^a^
S2214‐1	NA	NA	NA	NA	32	NA	NA	1536
S2214‐2	1270	1600	1086	2005	92	100	25–28	1501

Abbreviation: NA, Nonavailable.

^a^ Weight given with frozen content (blood in heart and stomach content in the stomach).

A complete parasitological investigation was performed on the blubber and the respiratory tracts. After removal, the entire blubber of the animals was thoroughly searched by making incisions from fascia to skin every 0.5–1.0 cm (Figure 4), resulting in the recovery of three cysts from the male, each containing a plerocercoid of *Clistobothrium* sp. (Cestoda: Phyllobothriidea; all were located posterior to the dorsal fin insertion, two on the right side and one on the left side). These are putatively identified as *Clistobothrium delphini* (Bosc, 1802) based on host and site of infection. However, morphological identification and molecular confirmation were not possible due to the poor state of the specimens and the DNA being degraded.

**FIGURE 4:**
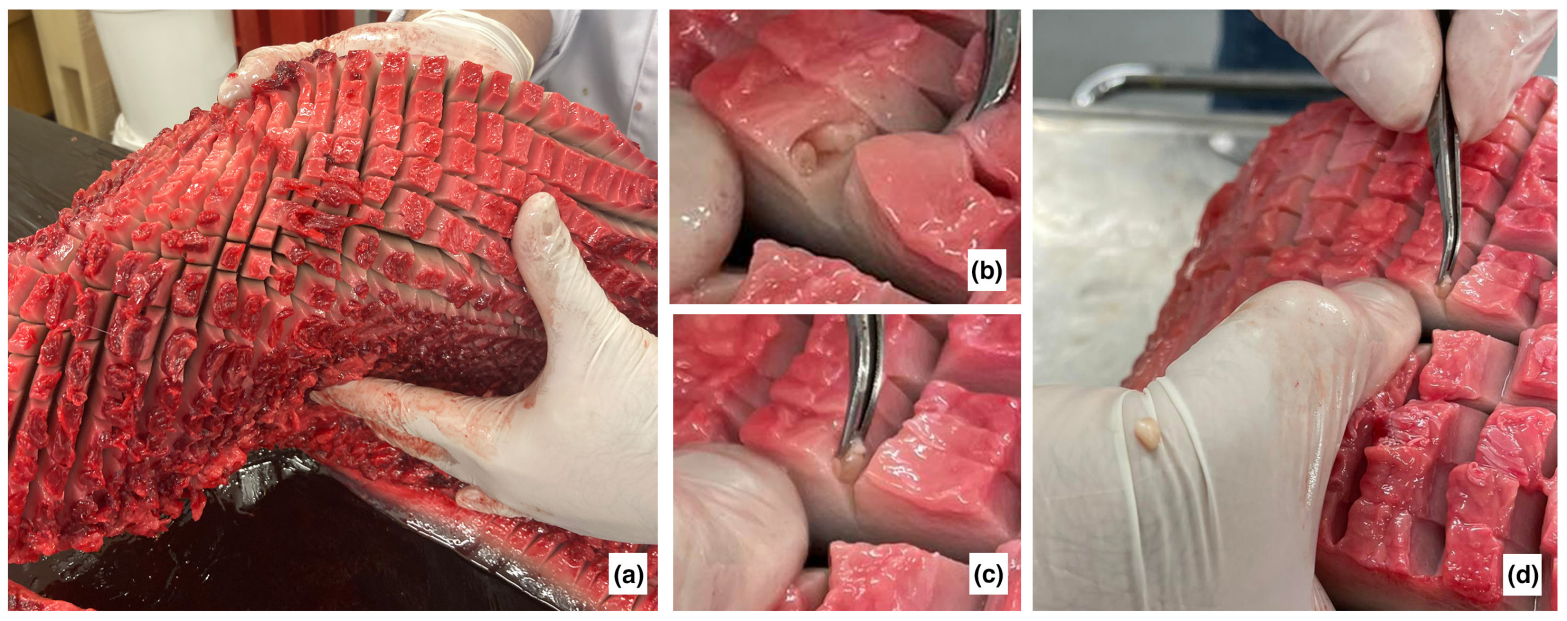
Pictures of the parasite investigation conducted during the necropsy of S2214‐2. (a) large piece of blubber with longitudinal and perpendicular incisions made every 0.5–1 cm on the whole blubber thickness: (b) discovery of cysts and (c) and (d) recovery of plerocercoid of *Clistobothrium* sp.

A parasite investigation was also conducted on the surface of all organs as well as the stomach wall of both animals and the intestine of S2214‐2, yielding no parasites. Furthermore, no hard structures, that is, cephalopod beaks or fish otoliths, were recovered from the intestine.

Viral infections were investigated by use of swab pins (FLOQSwabs, Coban) collecting samples from brain, bronchi, and lungs of both animals. They were placed immediately into the 3 mL UTM‐RT transport medium (Coban). Tubes were vortexed, centrifuged, and the supernatants were used directly for RNA extraction (RNeasy (Qiagen)). The RNA was tested in Real‐time RT‐PCR (RT‐qPCR) for detection of influenza A virus (IA). For pan‐influenza A virus, a RT‐qPCR assay targeting the M‐gene was used according to Hoffmann et al. (2010). The samples were also tested in pan‐H5 and pan‐N1 subtyping RT‐qPCR (Hassan et al., 2022).

Highly pathogenic avian influenza viruses (HPAIVs) of type H5N1 clade 2.3.4.4b has been spreading widely in wild and domestic birds since 2020. This subtype of influenza A virus has also been detected in several terrestrial and marine mammal species (World Organization for Animal Health 13‐2‐2023; https://www.woah.org/en/statement‐on‐avian‐influenza‐and‐mammals/). In this study, all the brain, bronchial and lung swabs tested IA‐negative using three different RT‐qPCR targets (e.g., primers and probes specific for universal influenza A virus M, H5, and N1 gene detection).

The stomach contents of both individuals were weighed and examined for species identification of prey remains. The only organic material found in both stomachs was algae. Each algae species fraction was individually weighed (Table 5). A large plastic fragment (Figure 5) was recovered from the male's stomach (S2214‐2).

**TABLE 5:** Composition of stomach contents of Risso's dolphin specimens S2214‐1 and S2214‐2, in weight and percentage for each algal species identified.

Species	S2214‐1		S2214‐2	
	Weight (g)	%	Weight (g)	%
*Ascophyllum nodosum*	10.43	46		
*Fucus vesiculosus*	11.92	53		
*Fucus distichus*			139.58	72
*Pylaiella littoralis*			52.91	27
Other^a^	0.30	1	2.54	1
Total weight (g)	22.65	100	195.03	100

^a^ Including gravel.

**FIGURE 5:**
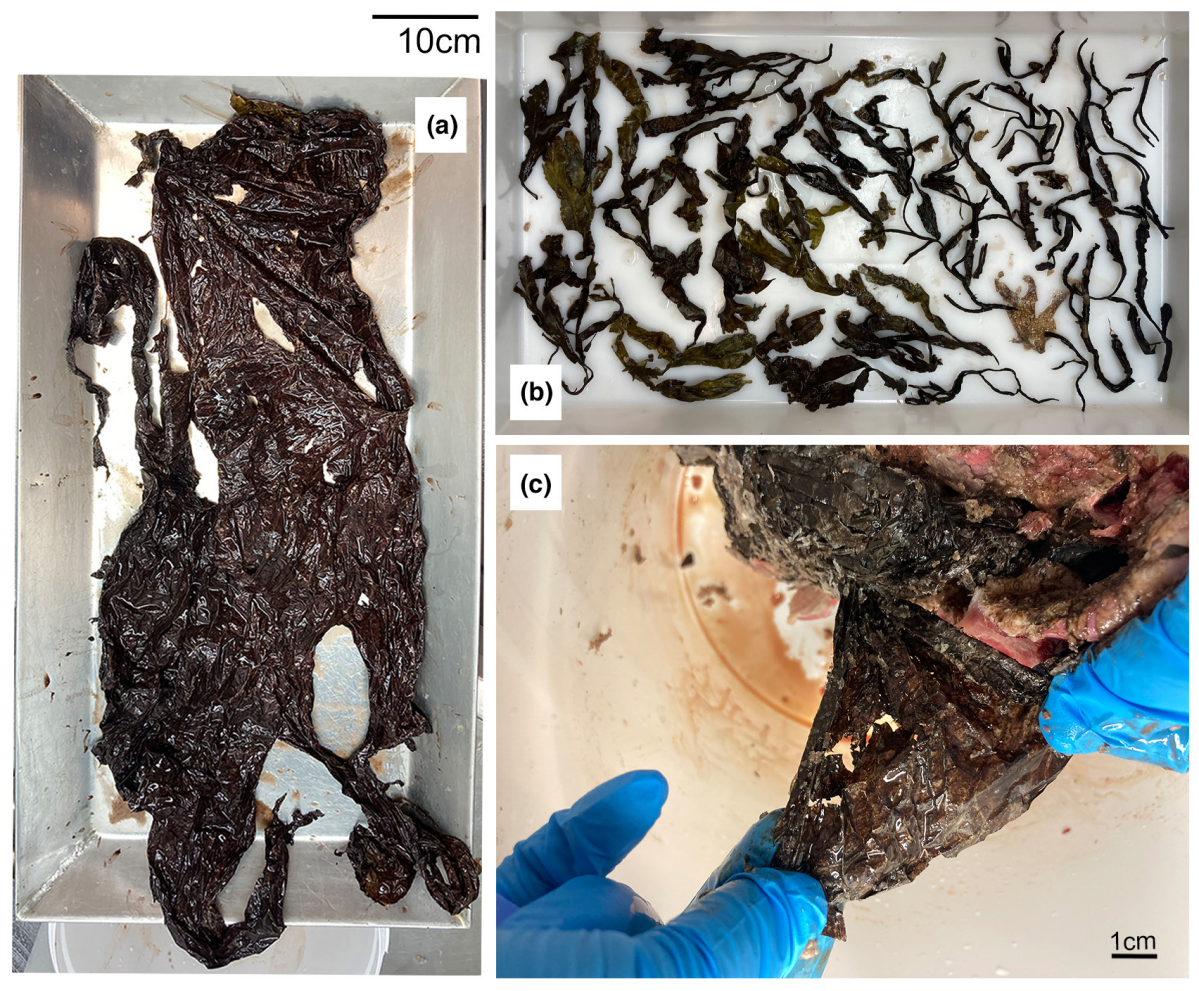
Pictures showing parts of the stomach contents of the stranded male Risso's dolphin S2214‐2: (a) and (c) a plastic bag retrieved from the stomach; (b) examples of the largest algae (mostly fucoid) retrieved from the stomach. A scale of 10 cm is indicated for (a) and (b), while a scale of 1 cm is indicated in (c).

The presence and number of teeth were recorded. The female had no visible teeth at the time of necropsy. However, two teeth were found and retrieved from the jaw bones when cleaned. Seven teeth from the lower jaw were collected from the male during necropsy, three on the left side and four on the right. They were collected for future age determination.

The skeletons of S2214‐1 and S2214‐2 were preserved for conservation and later exhibition at the Icelandic Institute of Natural History (Figure 6).

**FIGURE 6:**
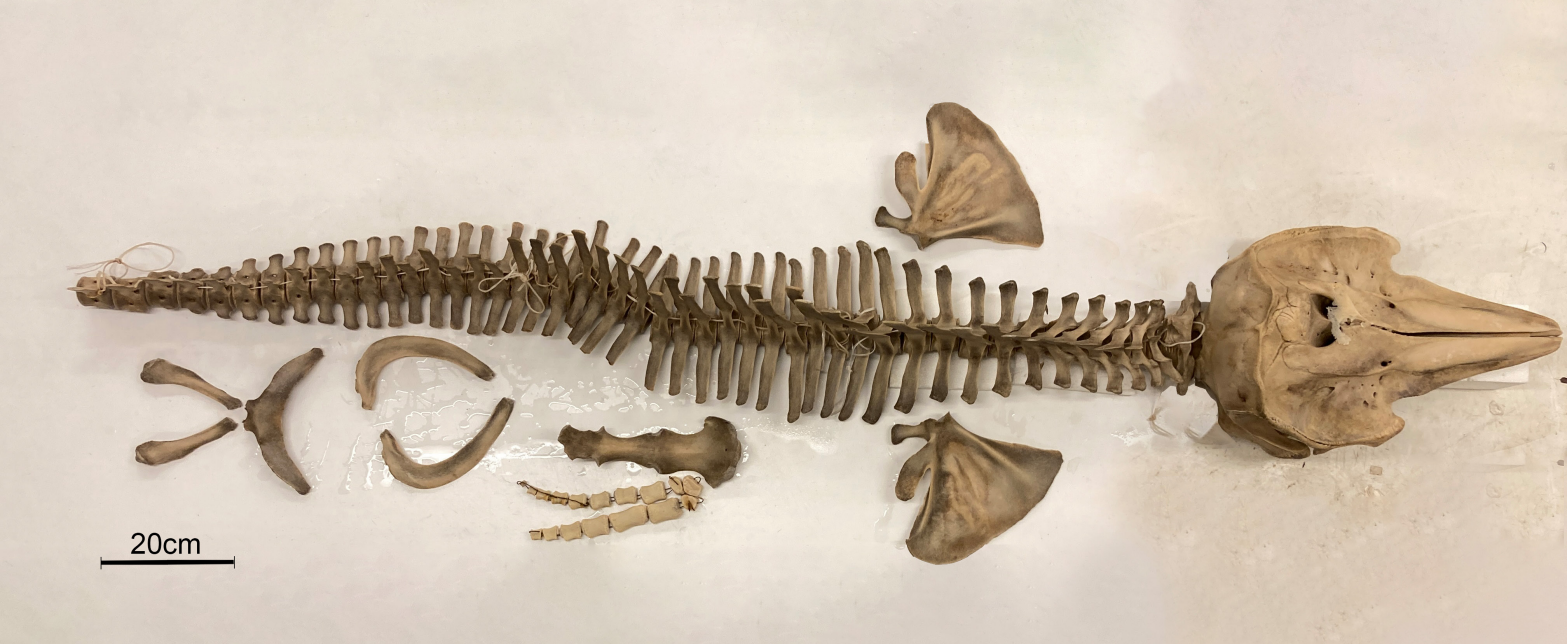
Reconstructed Risso's Dolphin's skeleton after being preserved by the Icelandic Institute of Natural History.

The storyline of these two Risso's dolphin strandings suggests that these individuals were traveling together as a pair. The juvenile male (S2214‐2; Figure 2b) stayed in the vicinity of the dead female's body (S2214‐1; Figure 2a), even when it had washed ashore. Based on their skin stage ranking (Hartman et al., 2015), the female and male were identified as an adult and juvenile, respectively (Figure 2). The female length is above the lower limits of length at sexual maturity listed by Perrin and Reilly (1984) and is in accordance with measurements reported for the smallest mature female of 277 cm in the eastern North Atlantic (Bolch et al., 2012). The male size, together with its testis weight of 53 g (Table 4), suggests that this is a juvenile of more than 2 years (Bolch et al., 2012). This adult‐juvenile pair could potentially be a mother and her offspring. Mother and offspring are known to remain together until the young reach puberty and become subadult (Hartman et al., 2008). Future age determination using the teeth collected should help resolve this question, along with genetic analyses to determine relatedness. However, it is not rare in delphinids to see a pair of two unrelated individuals composed of a mature female and a juvenile (Amano & Miyazaki, 2004; Hartman et al., 2008).

Based on BCS estimates, the condition of both individuals was very poor and emaciated (Table 1). This poor condition was supported by blubber thickness measurements, with as low values as 1.0–1.5 cm for both animals in certain areas. These extremely low body conditions suggest that these animals may have been in poor health status for an extended period of time. The analyses of the stomach contents support a lack of recent feeding activity. In both cases, no remains of the traditional prey of Risso's dolphin (squid and other cephalopods; Luna et al., 2021) were found in the stomachs (Figure 5, Table 5) and in the intestines. Instead, the only organic contents found were algae. The species of fucoid algae, found in the stomachs of both individuals (Table 5) are common in the rocky intertidal zone around Iceland as well as in the whole North Atlantic (Ingólfsson, 2006). *Ascophyllum nodosum* and *Fucus vesiculosus* can be found over the entire intertidal zone while *Fucus distichus* is usually confined to the lower parts. The stomach of the juvenile male contained only *F. distichus*, but we cannot rule out that *F. vesiculosus* was ingested by that individual much earlier and was, therefore, too digested to be identified. The lower digestion state of the stomach contents from the juvenile male can be explained by the occurrence of later feeding events, or by the presence of the large plastic fragment that may have prevented the digestion of the algae.

To the best of our knowledge, this sighting and stranding event of Risso's dolphins in Icelandic waters is the first observation of the species in this region of the Northeast Atlantic. While little is known of Risso's dolphin movements in the North Atlantic, most sightings in this area occur on the European continental shelf edge (Baird, 2009; Jefferson et al., 2014; Figure 1). Risso's dolphins are deep divers, diving frequently to depths between 400 and 1000 m in search of food (Baird, 2009; Jefferson et al., 2014). In the Northeast Atlantic, Risso's dolphins occur over the continental shelf edge but not in deeper waters (Bolch et al., 2012). Iceland is separated from the European continental shelf by either the Norwegian basin to the east or the Icelandic basin to the south, both exhibiting depths well over 2000 meters. This unusual sighting suggests either that these individuals were lost or that their distribution range may be shifting toward Icelandic waters, as previously suggested by Bolch et al. (2012). The very poor body condition of both individuals, as indicated by their BCS classification (Joblon et al., 2014), girth, and blubber thickness measurements, as well as the lack of remains of their preferred prey in their stomach, suggests that these individuals had not fed for an extended period of time. Unidentified illness may have prevented their ability to feed. From the presented data, collected during the necropsies, nothing indicated that the cause of death is something other than exhaustion of lost and starving individuals. However, organ analyses may, in the future, aid in determining any illnesses that were not easily identified during the necropsy.

Risso's dolphins tend to prefer tropical or mid‐temperate waters of the continental shelf between 30° N and 45° N of latitude but may occur across the ocean basin from 64° N and 46° S. The two individuals reported here were found on the northern coast of Iceland (> 66° N) where cold waters predominate (temperature < 7°C, Ólafsdóttir & Kennedy, 2022), suggesting that these individuals may have been outside their preferred habitat and thermal tolerance. Future steps in attempting to understand the origin of these individuals will include genetic or photo‐identification comparisons to other populations of the Atlantic or the Mediterranean Sea.

## AUTHOR CONTRIBUTIONS

**Valérie Chosson:** Conceptualization (lead); formal analysis (lead); investigation (lead); methodology (equal); resources (equal); writing – original draft (lead); writing – review and editing (equal). **Haseeb S. Randhawa:** Formal analysis (equal); funding acquisition (lead); investigation (equal); methodology (equal); resources (equal); writing – review and editing (equal). **Guðjón M. Sigurðsson:** Investigation (equal); writing – review and editing (equal). **Sverrir D. Halldórsson:** Resources (equal); writing – review and editing (equal). **Þorvaldur Þ. Björnsson:** Formal analysis (equal); investigation (equal); resources (equal). **Vilhjálmur Svansson:** Formal analysis (equal); investigation (equal); resources (equal); writing – original draft (equal). **Sandra M. Granquist:** Writing – review and editing (equal). **Karl Gunnarsson:** Formal analysis (equal); writing – review and editing (equal). **Filipa I. P. Samarra:** Formal analysis (equal); investigation (equal); writing – review and editing (equal). **Christophe Pampoulie:** Conceptualization (equal); funding acquisition (lead); resources (equal); writing – review and editing (equal).

## FUNDING INFORMATION

This study has received funding from the European Union's Horizon 2020 research and innovation programme under grant agreement No 817806 (SUMMER project). HSR received funding through a grant from the Eggerts fund (Eggertssjóður), hosted at the University of Iceland, and a Preparatory grant (Sóknarstyrkir) from the Icelandic Research Fund (Rannís).

## CONFLICT OF INTEREST STATEMENT

The authors declare no competing interests.

## Data Availability

Data and materials are available upon request to the first author. Mitochondrial sequences for the identification of animals are available in NCBI under the accession numbers OR381458 and OR381459. Data available on request due to privacy/ethical restrictions.
